# Imaging Features of Main Hepatic Resections: The Radiologist Challenging

**DOI:** 10.3390/jpm13010134

**Published:** 2023-01-10

**Authors:** Carmen Cutolo, Roberta Fusco, Igino Simonetti, Federica De Muzio, Francesca Grassi, Piero Trovato, Pierpaolo Palumbo, Federico Bruno, Nicola Maggialetti, Alessandra Borgheresi, Alessandra Bruno, Giuditta Chiti, Eleonora Bicci, Maria Chiara Brunese, Andrea Giovagnoni, Vittorio Miele, Antonio Barile, Francesco Izzo, Vincenza Granata

**Affiliations:** 1 Department of Medicine, Surgery and Dentistry, University of Salerno, 84084 Salerno, Italy; 2 Medical Oncology Division, Igea SpA, 80013 Napoli, Italy; 3 Division of Radiology, Istituto Nazionale Tumori IRCCS Fondazione Pascale—IRCCS di Napoli, 80131 Naples, Italy; 4 Department of Medicine and Health Sciences “V. Tiberio”, University of Molise, 86100 Campobasso, Italy; 5 Division of Radiology, Università degli Studi della Campania Luigi Vanvitelli, 80127 Naples, Italy; 6 Department of Diagnostic Imaging, Area of Cardiovascular and Interventional Imaging, Abruzzo Health Unit 1, 67100 L’Aquila, Italy; 7 Italian Society of Medical and Interventional Radiology (SIRM), SIRM Foundation, Via della Signora 2, 20122 Milan, Italy; 8 Department of Medical Science, Neuroscience and Sensory Organs (DSMBNOS), University of Bari “Aldo Moro”, 70124 Bari, Italy; 9 Department of Radiology, University Hospital “Azienda Ospedaliera Universitaria delle Marche”, Via Conca 71, 60126 Ancona, Italy; 10 Department of Clinical, Special and Dental Sciences, University Politecnica delle Marche, Via Conca 71, 60126 Ancona, Italy; 11 Department of Emergency Radiology, University Hospital Careggi, Largo Brambilla 3, 50134 Florence, Italy; 12 Department of Applied Clinical Sciences and Biotechnology, University of L’Aquila, 67100 L’Aquila, Italy; 13 Division of Epatobiliary Surgical Oncology, Istituto Nazionale Tumori IRCCS Fondazione Pascale—IRCCS di Napoli, 80131 Naples, Italy

**Keywords:** hepatic resections, liver, diagnosis, postoperative complications

## Abstract

Liver resection is still the most effective treatment of primary liver malignancies, including hepatocellular carcinoma (HCC) and cholangiocarcinoma (CCA), and of metastatic disease, such as colorectal liver metastases. The type of liver resection (anatomic versus non anatomic resection) depends on different features, mainly on the type of malignancy (primary liver neoplasm versus metastatic lesion), size of tumor, its relation with blood and biliary vessels, and the volume of future liver remnant (FLT). Imaging plays a critical role in postoperative assessment, offering the possibility to recognize normal postoperative findings and potential complications. Ultrasonography (US) is the first-line diagnostic tool to use in post-surgical phase. However, computed tomography (CT), due to its comprehensive assessment, allows for a more accurate evaluation and more normal findings than the possible postoperative complications. Magnetic resonance imaging (MRI) with cholangiopancreatography (MRCP) and/or hepatospecific contrast agents remains the best tool for bile duct injuries diagnosis and for ischemic cholangitis evaluation. Consequently, radiologists should be familiar with the surgical approaches for a better comprehension of normal postoperative findings and of postoperative complications.

## 1. Introduction

In the recent years the number of liver resections is globally rising, according to the increase in occurrence of primary and metastatic cancers [1,2,3,4]. Liver resection is still the most effective treatment of primary liver malignancies, including hepatocellular carcinoma (HCC) and cholangiocarcinoma (CCA), and of metastatic disease, such as colorectal liver metastases [5,6,7,8,9,10].

In the field of liver resection, according to the Brisbane 2000 terminology, liver resections could be classified as anatomic and not anatomic resection [10]. The type of liver resection (anatomic versus non anatomic resection) depends on different features, mainly on the type of malignancy (primary liver neoplasm versus metastatic lesion), size of tumor, its relation with blood and biliary vessels, and the volume of future liver remnants (FLT) [10]. Knowledge of the liver resection main type is necessary for the radiologist to recognize the radiological common features of post-operative findings, and to identify the possible postoperative complications.

Ultrasonography (US) is the first-line diagnostic tool to use in post-surgical phase. However, computed tomography (CT), due to its comprehensive assessment, allows a more accurate evaluation and more normal findings than the possible postoperative complications. Magnetic resonance imaging (MRI) with cholangiopancreatography (MRCP) and/or hepatospecific contrast agents remains the best tool for bile duct injuries diagnosis and for ischemic cholangitis evaluation [6].

The purpose of this narrative review is to report the main liver resection, focusing on the definition of anatomic versus non anatomic resection, and on the main regular postoperative radiological features.

### Type of Resection

Hepatectomies can be classified as anatomic and non anatomic resections.

## 2. Anatomic Liver Resection

Anatomic liver resection is defined as the complete removal of the liver parenchyma confined within the responsible portal territory. The portal ramifications define anatomical portions of the liver. In particular, the first order division of portal ramification defines the “hemi-liver”, the second order division defines the “section”, while the third order division defines the segment.

**Figure 1:**
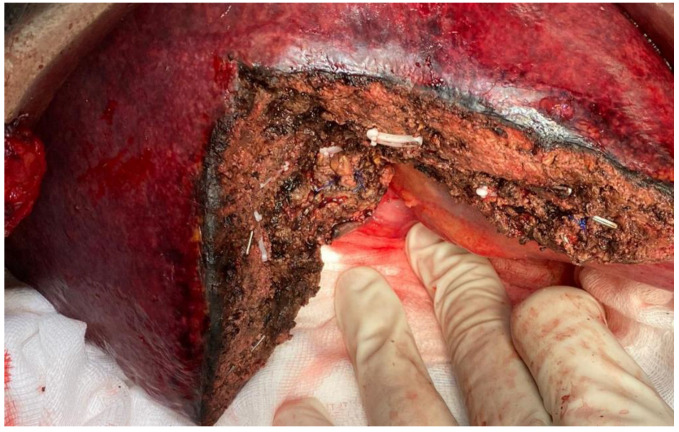
Anatomical bisegmentectomy of VI–VII in patient with metastases from kidney cancer.

**Figure 2:**
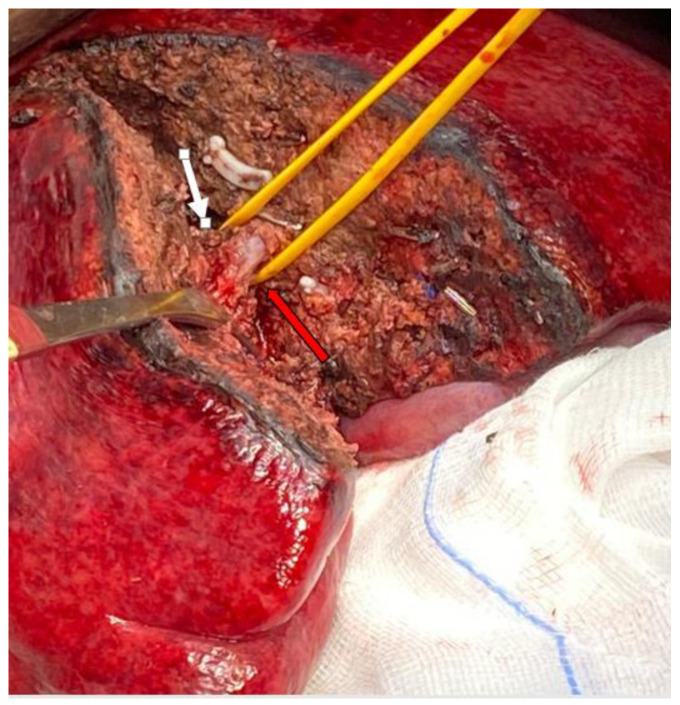
Anatomical segmentectomy of VI in colorectal liver metastasis (white arrow). Portal vein branch of VI–VII (red arrow).

Therefore, according to the Brisbane classification, anatomic liver resection is defined as segmentectomy (Figure 1 and Figure 2), sectionectomy, sectorectomy, hemiepatectomy, and trisectionectomy [10].

The term anatomic segmentectomy is utilized to describe the surgical approach that determine the elimination of a portion of liver parenchymal which correspond to a Couinaud segment. Otherwise, with “subsegmentectomy”, we identify a surgical approach to determine the partial removal of liver tissue within the portal territories of less than a Couinaud’s segment [11].

Anatomic sectionectomy corresponds to the comprehensive elimination of tissues of the second order portal venous branches. these approaches are classified considering the eliminated section. For example, the right anterior is the elimination of the right anterior, including segments 5 and 8, while the right posterior is the elimination of the right posterior, including segments 6 and 7.

The surgical procedure that causes the elimination of the liver to the right of the middle hepatic vein is defined as right hepatectomy. This resection includes segments 5, 6, 7, and 8. When in addition to right hepatectomy, segment 4 is resected, we obtained an extended right hepatectomy, also named right trisectionectomy.

With regard to the elimination of the liver parenchyma to the left of the middle hepatic vein, this approach is the left hepatectomy and includes the segments 2, 3, and 4. For extended left hepatectomy, also named as left trisectionectomy, the additional removal of segments 5 and 8 is intended.

## 3. Non Anatomic Liver Resection

**Figure 3:**
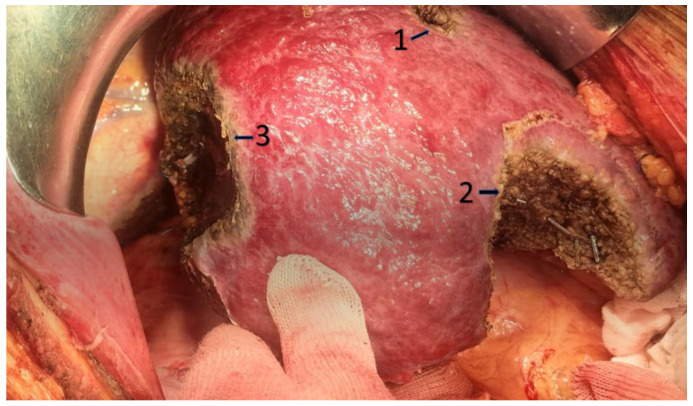
Multiple parenchymal sparing resections in patient with metastases from colorectal cancer. 1: Atypical resection of seg V; 2: Atypical resection of seg V-VI and 3: Atypical resection of segment VII.

**Figure 4:**
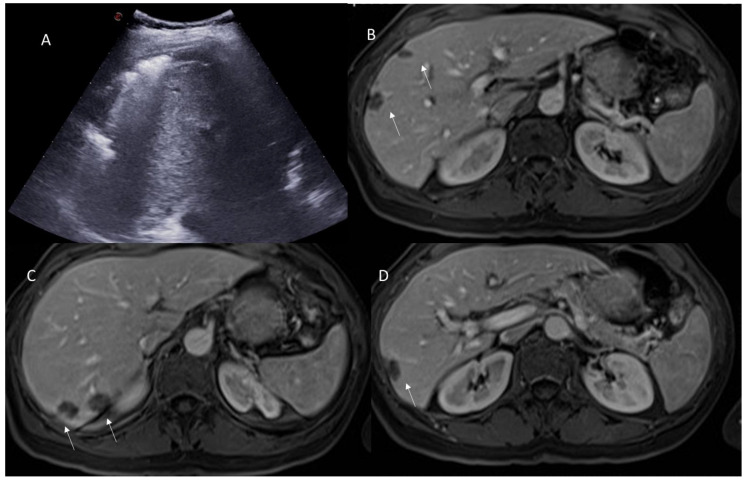
US (**A**) assessment and CT (**B**–**D**) assessment of Figure 3, patient. On portal phase of contrast medium evaluation, arrows ((**B**): V seg; (**C**): VII seg and (**D**): VI seg) show typical fluid collection due to surgical procedure.

The surgical procedure known as parenchyma-sparing hepatectomy (PSH) is a limited non anatomical liver resection (Figure 3 and Figure 4). In contradiction of anatomic resections, which involve systematic anatomical hepatic resection, this strategy allows to spare a certain future remnant liver volume and minimize surgical stress and operative risks [12,13,14,15].

The PSH procedure should allow an R0 resection (corresponds to resection for cure or complete remission) with negative surgical margins. Compared to the anatomical resection (AR) approach, it is more technically challenging, since anatomic landmarks, as lobar or sectorial vessels, are not used as a guide to identify the resection margins. Therefore, intraoperative ultrasound plays a key role during PSH, allowing the identification of lesions, the relationship of the lesion with the vital structures (e.g., hepatic veins, portal structures), and the parenchymal transection plan [16].

Although the cut off to obtain negative margins has usually been 1 cm, the introduction of new chemotherapeutic agents has allowed the reduction of this margin at 1 mm, in colorectal metastases (CLM), with good oncological results [17]. Moreover, it has been demonstrated that these new treatments are responsible for similar overall survival (OS) among R0 patients and patients with microscopic positive margins (R1) [18,19]. Hence, surgical resection should also be performed in R1 subgroup patients [18,19].

In addition, according to the new surgical oncology group suggestions, in the absence of extrahepatic disease, the only limits to surgical treatment of CLM patients is correlated to the post procedural liver parenchymal that should be sufficient to prevent liver failure (PLF) [20].

**Figure 5:**
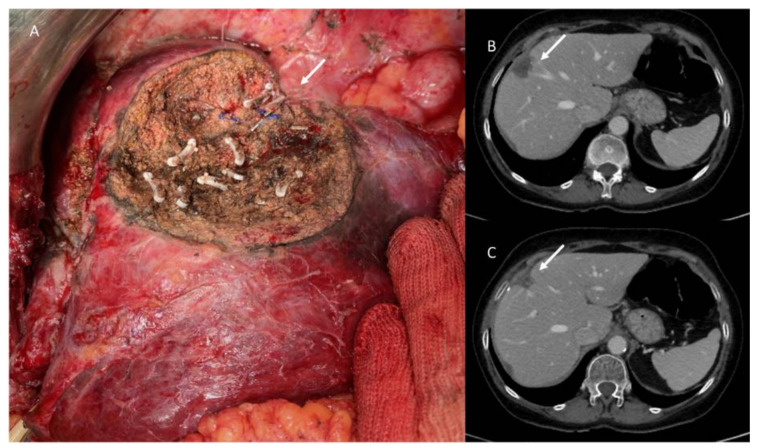
In (**A**), atypical liver resection of lesion located in V-VIII segment in patient with metastases from colorectal cancer. In (**B**,**C**) CT post treatment evaluation. At discharge, arrow shows fluid collection; at 3 month CT follow-up, arrow shows scar tissue (**C**).

Thanks to the use of the intraoperative US, it is possible to detach lesions from major vessels and to perform accurate flow analysis, preserving communicating vessels among main hepatic veins that should assure an adequate outflow to the liver even after main hepatic vein resection [21,22,23,24]. The use of this new procedural technique opened the opportunity to achieve new liver resection sub-types, which could substitute conventional hepatectomies (Figure 5), considering the patient characteristics, to obtain a more personalized treatment [24,25,26,27,28,29,30].

Among all the complex parenchymal sparing strategies, we cite systematic extended right posterior sectionectomy [26], mini upper transversal hepatectomy [24,25,26,27,28,29,30,31], right upper transversal hepatectomy [28], left upper transversal hepatectomy [24], total upper transversal hepatectomy [24], mini mesohepatectomy [27], liver tunnel [24,30], and liver tunnel extended to segment 4s [24,30].

### 3.1. Two Stage Hepatectomy and ALLPS

Two stage hepatectomy and associating liver partition with portal vein ligation for staged hepatectomy (ALPPS) are two strategies of treatment for liver disease in patients with insufficient liver function [32].

With regard to two stage hepatectomy, this approach includes two phases: In the first, to obtain hypertrophy of future liver remnant (FLR), the surgeons and/or radiologists perform a surgical ligation or radiological embolization of the portal vein, so that, the redistribution of portal flow induces parenchymal hypertrophy. In the second phase, the surgical approach is performed to remove target lesions.

The ALPPS includes parenchymal splitting and portal vein ligation in the first stage [33]. Complete redistribution of portal blood flow causes FLR hypertrophy. Although this approach increases the patient number that can be subjected to liver resection, it is correlated with significant operative morbidity and mortality [34].

### 3.2. Treatment and Imaging Assessment

Imaging, with regard to hepatic resection, should be employed in different phases: staging, treatment planning, intra-treatment evaluation, and treatment response assessment, which includes technical success, treatment efficacy, and complications [35,36,37,38,39,40,41,42,43,44,45,46,47,48,49,50,51,52,53,54,55,56,57,58,59,60].

With regard to “technical success”, this is due to the ability of treating the lesion according to the procedure [61], while “technique efficacy”, that should be distinguished from “technical success”, is the “complete resection”, also named R0, of a macroscopic lesion [61]. Complications, defined as any unpredicted deviation from a procedural course, and/or adverse events, recognized as any possible damage correlated to the procedure, should be evaluated according to standardized classification, as the Common Terminology Criteria for Adverse Events and the Clavien–Dindo classification [62]. In addition, any adverse events should be classified considering the severity and the time of occurrence (e.g., during, in post-procedural phase, or late) [61,62,63,64].

Different diagnostic techniques may be utilized, alone or as a multimodality approach [65,66,67,68,69,70,71,72,73,74,75,76,77,78,79,80]. Computed tomography (CT) and magnetic resonance imaging (MRI) are the main imaging tools employed during the pre-procedural phase in order to evaluate liver lesions [81,82,83,84,85,86,87,88,89,90,91,92,93,94,95,96,97,98] and in the surveillance phase to assess the efficacy and the safety [99,100,101,102,103,104,105,106,107,108,109,110,111,112,113,114,115,116,117,118,119,120,121].

Ultrasound assessment, also with the employment of contrast medium (CEUS), is a relatively new tool, utilized for problem solving during treatment phases and surveillance [122,123,124,125,126,127,128,129,130,131,132], although the critical point of interest is due to the possibility of real time procedure efficacy assessment. In fact, CEUS allows to detect perfusion change during the procedure, and bearing in mind the higher temporal resolution and the possibility of repeating this diagnostic exam several times in a short period, it is a secure and cost-effective tool for treatment outcome assessment [130,131,132].

Usually, US is the first tool employed during the post-surgical phase to assess abdominal complications and to evaluate treatment efficacy. CT with contrast agents, normally, is utilized as a follow-up tool to evaluate efficacy and recurrence, while it is the first tool employed in emergency setting (e.g., major complications as posthepatectomy hemorrhage (PHH)) [35,36,37]. MRI with cholangiopancreatography sequences (MRCP) or with hepatospecific contrast agent (EOB-MRI) is the best modality for diagnosis of early postoperative bile duct injuries and ischemic cholangitis, while during follow-up this tool is a problem solver for indeterminate liver lesions, e.g., new lesions versus abscesses [35,36,37].

### 3.3. Post-Surgical Imaging Findings

The post-surgical radiological assessment should be distinguished early, during the first hours after surgery, with a follow-up at the time of discharge and an oncological follow-up taking into account the main guidelines in relation to the type of cancer treated [133,134,135].

Normally, a diagnostic assessment, during the first hours after surgical procedure is only required in case suspected complications, such as a bleeding or biliary lesion [136,137,138,139], while at the time of discharge US assessment is required. CT or MRI should be performed to confirm complications.

### 3.4. Discharge Assessment

In this phase, radiologist should evaluate: (a) the presence of free fluid, (b) the state of the surgical margins and the remnant liver parenchyma, (c) vascular evaluation (portal vein, hepatic veins, and hepatic arterial branches) as well as any other sites subjected to resection [140,141,142,143,144,145,146,147].

At US assessment, free fluid may present a hypoechoic collection in the posterior recesses or, in the presence of blood, inhomogeneously iso-hypoechoic or hyperechoic [140,141,142,143,144,145,146,147]. In this case, a clinical laboratory evaluation is mandatory, considering the proposal of the International Study Group of Liver Surgery (ISGLS) [148], in which we found a novel definition and staging of PHH. According to these guidelines, PHH is defined as a drop in haemoglobin level >3 g/dL compared to the post-operative baseline level (i.e., haemoglobin level immediately after surgery), with three grades of severity (A-B-C), depending on the therapeutic strategy required [148].

**Figure 6:**
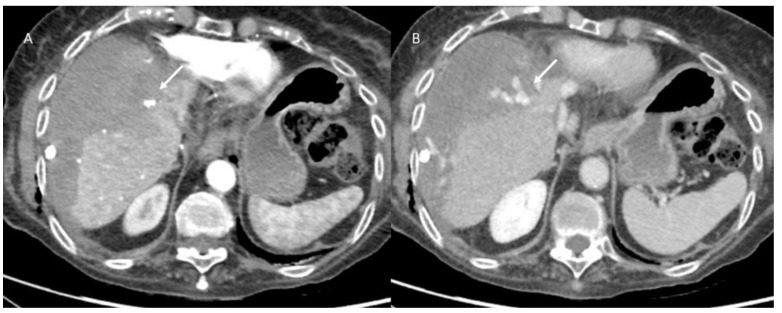
Active bleeding. During arterial CT assessment (**A**) and portal evaluation (**B**), arrow shows active overflow of contrast material.

To assess active bleeding and to identify bleeding causes, a multiphasic CT evaluation is mandatory (Figure 6) [149,150,151,152,153,154]. During pre-contrast CT evaluation, a blood collection with an attenuation of 30–45 HU could be found [155,156,157,158,159,160,161,162,163,164,165]. The presence of the sentinel clot sign, with attenuation values of 45–70 HU, can help identify the site of bleeding. During arterial CT assessment, the active overflow of contrast material is suggestive of arterial bleeding, while low-flow bleeding is detected during venous CT phase [155,156,157,158,159,160].

At US assessment, surgical margins may appear hyperechoic compared to surrounding liver parenchymal. In addition, the radiologist should evaluate the presence of post-surgical fluid collections. These entities may be due to the presence of haematomas (50%), bilomas (25%) and abscesses (25%) [35].

Hematoma may appear as a biconvex or growing intraparenchymal lesion, with suprafluid echogenicity at US or density at unenhanced CT (between 50 and 60 HU) [35,161,162,163,164].

**Figure 7:**
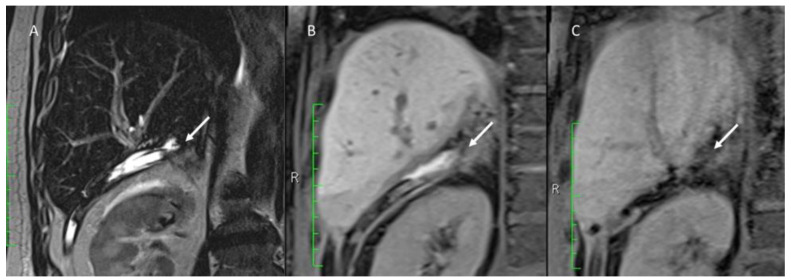
Post-surgical MRI assessment. In (**A**) T2-W sequences shows (arrow) fluid collection. In (**B**), On EOB-phase, bile leak is detected as an active overflow of contrast material outside biliary tree and inside the fluid collection (arrow). In (**C**) EOB-phase post bile leak treatment evaluation, no overflow of contrast medium.

Biloma is an encapsulated collection of bile outside the biliary tree [35]. At US assessment, it may appear as simple fluid collections [35]. If a biloma is supplied, we have a bile leaks. A definition for bile leak was standardized by the ISGLS [165]. The leakage may be due to an incompetent bile-digestive anastomosis or a bile ducts damage during the surgical procedure [166,167,168,169,170,171]. At US or CT examination, bile leaks may appear as a non-specific collection near the resection margins [165]. MRI with gadolinium- based hepatobiliary contrast agent (EOB-MRI) allows for proper site detection so as to classify the leakage sub-type (Figure 7) [161,162,163,164,165,166,167,168,169,170,171,172,173,174,175,176,177]. On EOB-phase, bile leak is detected as an active overflow of contrast agent outside the biliary tree and inside the fluid collection [35].

Air artefacts within a supra-fluid collection that do not show central perfusion at color Doppler assessment in patients with high grade fever is suggestive of an abscess [35]. CT confirms the diagnosis showing the typical features of a double target appearance, characterized by a central hypodense core of fluid surrounded by a hyperdense rim and a hypodense outer ring [35]. The use of haemostatic glues on the resective margins could mimic an abscess due to the presence of hypodense microbubbles [78]. An accurate clinical evaluation (absence of fever and inflammation indices) allows a correct diagnosis [78].

The radiological appearance of remnant liver parenchymal is complex and is correlated to the type of surgical procedure, the segment resected (ore more segment), the quality of residual parenchymal (cirrhotic or chemotherapy-induced steatohepatitis) [178]. A non-typical finding is the hepatitis due to treatment, that can be hepatocellular, cholestatic, or mixed [140,178]. During imaging assessment, it is possible to find hepatomegaly, perihepatic fluid, lymphadenopathy, and periportal edema [140]. The main feature is the gallbladder wall thickening or gallbladder fossa edema. On US assessment, typical findings are a parenchymal echogenicity decreasing with an increase of the portal vein conspicuity (known as “starry sky”) [178]. On CT or MRI evaluation, it appears as liver attenuation decreasing or diffuse hyperintensity in T2-weigthed (T2-W) sequences [140,178]. During contrast medium evaluation, a heterogeneous parenchymal enhancement, due to perfusion re-assessment, could be detected. Severe cholestatic hepatitis on MRCP appears as a decreasing of the tertiary bile ducts number [178].

Vascular assessment provides for the evaluation of the flow of the main arterial and venous branches [179,180,181,182,183,184,185,186,187,188,189]. Therefore, the first tool to use can be the color Doppler. In uncooperative patients or in doubtful cases, multi-phases CT is the best tool to use [35].

Clinically, vein thrombosis may be asymptomatic or may cause abdominal pain if the superior mesenteric vessels are involved due to bowel congestion or ischemia [158,190,191]. When not detected, collateral vessels will grow, and the patient will develop portal vein cavernous transformation [192]. Otherwise, arterial thrombosis may cause liver failure, sepsis, or abscess [160].

On US assessment, a limited thrombus is seen as an echogenic area within vessel, in the absence or with a slow portal flow on Doppler images. In addition, on color Doppler US, vein thrombosis is characterized by the loss of a triphasic waveforms pattern with a decrease in hepatic vein velocity and in portal flow [35,193]. On CT contrast study evaluation, arterial or portal thrombosis appears as an intraluminal filling defect of the hepatic artery or portal vein, during arterial and porta phase, respectively. Venous thrombosis could be intercepted on unenhanced CT as intraluminal hyperattenuating spots within the vessel [35]. In addition, during arterial phase and correlated to the compensatory augmentation of local arterial flow, it is possible to find a segmental enhancement (transient arterial hyperenhancement- THAD) of the tributary liver parenchyma [35].

### 3.5. Follow-Up Assessment

During the follow-ups scheduled in relation to the patient’s cancer history, the findings described at discharge change [140].

**Figure 8:**
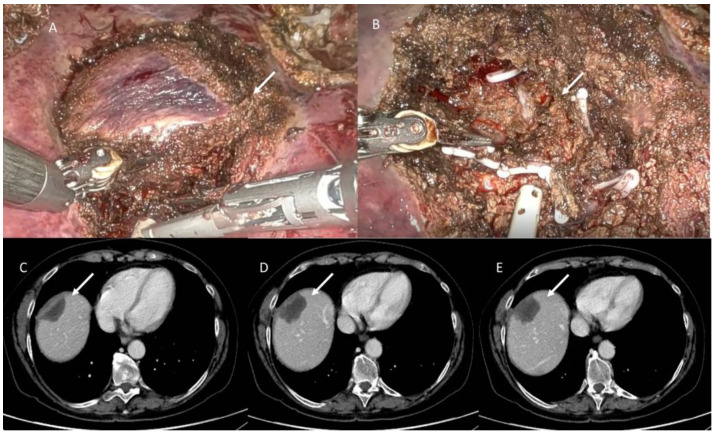
In (**A**,**B**) robotic atypical resection of lesion located in segment VIII. In (**C**–**E**), CT evaluation: arrows show fluid collection.

**Figure 9:**
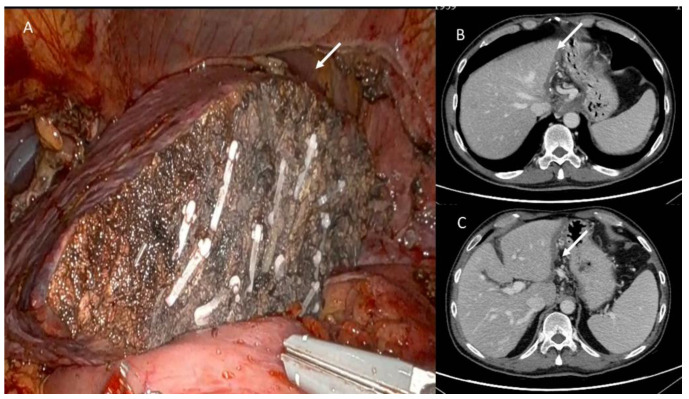
Robotic Anatomycal S2 segmentectomy in patient with HCC (**A**). In (**B**,**C**), CT assessment at 3 months follow-up: arrows show scar tissue.

In the patient with a functioning liver, free effusion disappears [140], the intra liver collections reduce in size (Figure 8), and they are replaced by scar tissue (Figure 9) [140].

This tissue should be correctly distinguished from new lesions, expressions of disease recurrence [140,194,195,196,197,198,199,200,201,202,203]. On US assessment, scar tissue is iso-hyperecoic without contrast enhancement during CEUS evaluation [140]. CT and MRI allow the proper lesions characterization [204,205,206,207,208,209,210,211,212,213], also thanks to the possibility of functional evaluation (diffusion weighted imaging and radiomics) [214,215,216,217,218,219].

With regard to remnant liver parenchyma, hepatitis is replaced by hepatic regeneration, so radiologist should know the type of surgical procedure to correctly localize new lesions [140]. Otherwise, the correct localization is possible with the identification of the main arterial and venous branches, and so as a biliary tree [140].

### 3.6. Two Stage Hepatectomy and ALLPS Assessment

With regard to two stage hepatectomy and ALLPS radiological evaluation, these procedures cause a selective portal vein occlusion to obtain a hypertrophy of future liver remnant [32,33,34]. So, in this context, the radiologist should evaluate vein thrombosis and the liver parenchymal compared to pre-treatment diagnostic study. However, the assessment of hepatic regeneration is a volumetric evaluation, and this is not correlated to the real parenchymal functionality [220,221,222].

## 4. Conclusions

The knowledge of the main type of liver resection is necessary for the radiologist to recognize the radiological common features of post-operative findings and identify the possible postoperative complications.

US is the first-line imaging examination during the postoperative monitoring. However, CT is of greater value for identifying normal findings after surgery, and the possible postoperative complications. MRI is the best modality for the diagnosis of early postoperative bile duct injuries and to assess recurrence.

## Data Availability

Data are reported in the manuscript.
